# Dosage of epidural morphine analgesia after lower abdominal cancer surgery: a randomized clinical trial among the older adults

**DOI:** 10.1186/s13741-025-00521-z

**Published:** 2025-05-06

**Authors:** Muhammad Shawqi, Sahar Abdel-Baky Mohamed, Essam Sharkawy, Diab Hetta

**Affiliations:** 1 Department of Anaesthesiology, South Egypt Cancer Institute, Assiut University, Assiut, Egypt; 2 Department of Anaesthesiology, Faculty of Medicine, Assiut University, Assiut, Egypt

**Keywords:** Epidural analgesia, Older adults, Cancer surgery, Post-operative pain, Pharmacokinetics-pharmacodynamics

## Abstract

**Background:**

Epidural morphine is considered one of the most potent drugs used for postoperative analgesia; however, its side effects are dose-related and exaggerated in elderly people. In this study, we aimed to determine which of three doses within that range (1.5 mg, 3 mg, or 4.5 mg) can provide adequate pain relief.

**Methods:**

A total of 102 patients were assessed for allocation into one of four groups to receive either placebo (group Morphine 0, *N* = 22), 1.5 mg of epidural morphine (Morphine 1.5, *N* = 22), 3 mg of epidural morphine (Morphine 3, *N* = 22), or 4.5 mg of epidural morphine (Morphine 4.5, *N* = 22) before skin incision, 24 h after surgery and 48 h after surgery. Cumulative intravenous IV-PCA morphine consumption, VAS pain scores, modified Ramsay Sedation Scores, nausea, vomiting, and pruritus were evaluated.

**Results:**

The VAS pain scores at activity of patients who received epidural morphine at doses of 3 mg and 4.5 mg were significantly lower than the placebo and 1.5 mg groups, VAS Score at 72 h was (2 ± 0.8) and (1.7 ± 1) vs (4.3 ± 1.1) and (4 ± 1) respectively, *p* value = 0.000. The mean total IV-PCA morphine consumption (mg) was significantly higher in patients who received received epidural 0.9% sodium chloride alone compared to 1.5 mg, 3 mg and 4.5 mg epidural morphine groups (38.1 ± 4.8 mg vs 27.2 ± 5.6 mg, 9.2 ± 3.5 mg, and 6.3 ± 3.3 mg respectively), *p* value = 0.000). However, the difference between the 3 mg and the 4.5 mg groups was not statistically significant in both of VAS scores and IV-PCA morphine consumption (P value > 0.05 for 3 mg vs. 4.5 mg).

Patients who received 4.5 mg of epidural morphine experienced a significant increase in the level of sedation, measured by the Ramsay sedation scale, in comparison with 1.5 mg, 3 mg and placebo epidural morphine groups in the first 24 h, the Scale for this group was (2.5 ± 0.5) vs (2.1 ± 0.2, 2.1 ± 0.2, and 2.2 ± 0.5 respectively); *p* value = 0.000. No relationship between postoperative nausea and the dosage of epidural morphine was found.

**Conclusion:**

Epidural morphine 3 mg as a bolus every 24 h with add on IV patient control analgesia (PCA) morphine, set to deliver 1.5 mg boluses on demand without background infusion with a lockout period of 45 min, could achieve effective and adequate analgesia lasting up to 72 h postoperatively without increasing in the level of sedation or other side effects in older adults after a lower abdominal cancer surgery.

**Supplementary Information:**

The online version contains supplementary material available at 10.1186/s13741-025-00521-z.

## Introduction:

Sixty-five-year-old and older patients represented 43% of the total inpatient hospital stays in the United States (Hall et al. 2007). The number of older adults who undergo surgery for cancer is increasing, and the management of their pain after surgery is challenging. Furthermore, how to protect organ function in the perioperative period appears to be the next research trend for the care of the elderly population (Chen et al. 2022). Uncontrolled postoperative pain in older adults results in longer hospitalisation and rehabilitation. Postoperative pain, especially after abdominal surgery, affects pulmonary function by hindering coughing and deep breathing, thus reducing the elimination of secretion from the respiratory tract, decreasing vital lung and functional residual capacity, leading to atelectasis, and respiratory infections.

In the US, among 1140 cases who underwent radical cystectomy between 1983 and 2008 in Norris Comprehensive Cancer Center, 185 cases (16%) of the patients aged more than 65 years were categorized as ASA I or II (Djaladat et al. 2014). In addition, among 1166 cases who underwent primary surgical treatment for endometrial cancer at the Department of Obstetrics and Gynecology, Helsinki University Hospital, between 1 January 2007 and 31 December 2013, 527 (45%) aged above 60 years were categorized as ASA I or II (Kolehmainen et al. 2019). In the republic of Korea, 316 cases with mean age of 66 years were reported to be ASA I or II (91%) out of 480 patients with upper tract urothelial carcinoma from four different institutions (Kang et al. 2017). Thoracic epidural analgesia (TEA) is recommended as a first-line pain treatment after major abdominal surgeries (Gustafsson et al. 2019; Chou et al. 2016). All epidural opioids, including morphine, have the advantage of producing analgesia without sympathetic or motor blockade, which leads to early mobilisation and stable blood pressure (Sultan et al. 2011; Brill et al. 2003) Morphine is the first reported opioid used in the epidural space and has been used for more than 40 years (Leon-Casasola and Lema 1996). Although many studies have been performed to evaluate the dosage and analgesic effect of epidural morphine, we found no studies addressing this problem among the older adults who undergo lower abdominal cancer surgery such as bladder or uterine cancer.

Aging has a considerable influence on the dosage of opioids for perioperative pain management due to altered pharmacokinetics-pharmacodynamics (PK-PD). The parent-metabolite relationship significantly altered in older adults with renal dysfunction, making opioid overdose more likely (Mercadante and Arcuri 2004). The most common adverse effects in older patients with renal dysfunction is the increased risk of respiratory depression (Mercadante 2024). Compared with systemic administration, epidural morphine has the advantages of potent analgesia, a longer duration of action and some dose-sparing effects (Sultan et al. 2011). The main side effects of epidural morphine include, prolonged sedation and respiratory depression, itching, nausea and vomiting (Bonnet et al. 2010). The incidence of respiratory depression, early or delayed, may increase in older people with severe postoperative complications such as aspiration (Mann et al. 2000).

In current clinical practice, single boluses of epidural morphine analgesia are rarely delivered alone and routinely admixed with a local anaesthetic for fear of delayed respiratory depression. The recommended initial epidural morphine dose ranges from 3.5 mg to 7.5 mg daily for young patients. For older adults, it ranges from 1.5 mg to 4.5 mg per day within our institution for any abdominal surgery because of the greater sensitivity to epidural morphine in this age group (Martinez-Velez and Singh 2023).

In this study, we aimed to determine which of three doses within that range (1.5 mg, 3 mg, or 4.5 mg) is the optimal dose. An optimal dose of epidural morphine analgesia can be described as the dose that can both produce and maintain adequate analgesia with minimal dose-related side effects (Sultan et al. 2011).

## Materials and methods

This study is a parallel group, placebo-controlled, double-blinded randomised clinical trial. Institutional Ethics Committee approval was obtained from the Institutional Review Board (IRB), Assiut University Faculty of Medicine, Egypt, Approval No: 17101147. Written informed consent was obtained from each included patient before enrollment. The study was conducted according to the Consolidated Standards of Reporting Trials (CONSORT) statement. It was registered at clinicaltrials.gov with ID number NCT04316871 before the start of the study.

Patients were recruited from surgical oncology departments between March 2020 and June 2021. The inclusion criteria were men and women aged ≥ 60 years, American Society of Anaesthesiologists physical status (ASA) class I and II with normal cardiac, liver and kidney function with no history of preoperative local or distant metastasis, previous chemotherapy or severe anemia (Horvath et al. 2021), who were scheduled to undergo open lower abdominal cancer surgery via an infra-umbilical abdominal incision, such as total abdominal hysterectomy (TAH), radical cystectomy, or colectomy. Those 60 years of age and older were selected as older adults for two reasons. First, as a developing country, we followed the United Nations (UN) definition of an older population of 60 + years. Second, the life expectancy in Egypt is 72 years according to World Bank 2020 data (Life expectancy at birth, total (years) - World | Data n.d 2024). The exclusion criteria were patients with contraindications to neuraxial analgesia, including coagulopathy, infection at the site of the epidural catheter, high intracranial pressure (ICP), severe dehydration or preexisting neurological disorders such as multiple sclerosis (MS). In addition, we excluded patients with psychiatric disease that would interfere with the perception or the assessment of acute pain such as psychosis, schizophrenia, severe depression or anxiety disorder, bipolar disorder or any other serious mental illness. We also excluded patients who received analgesics other than our standardized postoperative Intravenous Patient-Controlled (IV-PCA) morphine protocol at any time after surgery, patients who were scheduled for laparoscopic surgery, or patients who were hypersensitive to epidural morphine or any drug used in the study. Additionally, patients who refused to enrol were not included in the study. The secondary exclusion criterion was any reason that resulted in a protocol violation.

The recommended level of epidural catheter placement is T9–T10 for mid and lower abdominal procedures (Chekol et al. 2020). The epidural catheters were inserted at T9 - 10 levels because the incidence of postoperative paralytic ileus under TEA was reported to be significantly lower if injected above T12 (Zingg et al. 2009). We assessed the delivery of three different doses of epidural morphine (1.5, 3, and 4.5 mg) to three independent groups compared to a placebo group. The diluted preservative-free epidural morphine studied doses were identical in appearance to be unrecognisable, so neither the patient nor the attending anaesthesiologist could identify the administered dose.

A blind observer nurse, who was not a part of the anaesthesia team or study team, followed up with the patients. The primary outcome measure was cumulative postoperative 72-h IV morphine consumption. The following secondary outcomes were assessed: (1) postoperative pain intensity at activity that causes discomfort, such as coughing (Mello et al. 2014), measured by Visual Analog Scale (VAS) (Myles et al. 2017) at (0, 2, 8, 16, 24, 36, 48 and 72 h) postoperatively, which was reported from 0 to 10 (where 0 = no pain and 10 = the worst pain ever), and all patients were taught how to evaluate their pain and how to use patient-controlled analgesia (PCA) the day before surgery; (2) sedation level assessed by the Modified Ramsay Sedation Score (Sessler et al. 2008), where awake levels were as follows: I = anxious, agitated, or restless; II = cooperative, oriented, and tranquil; III = response to command, and asleep levels were dependent on the patient's response to a light glabellar tap or loud auditory stimulus; IV = brisk response; V = a sluggish response; and VI = no response; and 3) assessment of side effects (nausea, vomiting, and pruritus) was scored on a scale of 0–2, where 0 was recorded if the side effect was absent; 1 if the side effect was minimal and did not require treatment; and 2 if the side effect was moderate or severe and required treatment. The 72-h score for each side effect was the sum of its scores at the eight points (0, 2, 8, 16, 24, 36, 48 and 72 h).

A standardised 72-h postoperative analgesia protocol of IV-PCA morphine was also delivered over these time intervals for all study groups. All patients used the IV-PCA and were instructed to maintain a VAS pain intensity ≤ 3 by using the PCA device. Postoperative analgesia was composed of IV-PCA morphine, set to deliver 1.5 mg boluses on demand, meaning that the VAS score of the patient was > 3, without background infusion with a lockout period of 45 min. All patients were instructed to maintain a postoperative VAS pain intensity ≤ 3 by using the PCA device, that enabled us to use IV-PCA morphine consumption as a surrogate indicator for the severity of postoperative pain at rest in our study. Instead, assessment of each activity that causes discomfort, such as coughing, respiratory exercises, manipulation by the health staff or procedures considered to be painful, is recommended (Mello et al. 2014).

Patients were randomised by using a table of random numbers concealed within a sealed envelope that was opened before the procedure to one of four groups to receive a study dose of four doses of epidural morphine (0, 1.5, 3, or 4.5 mg). The start dose was given immediately before skin incision followed by two subsequent bolus doses every 24 h postoperatively with background epidural infusion of 0.9% sodium chloride at a rate of 20 ml/hr until the removal of epidural catheter after 72 h postoperatively.

In the operating room, a venous line was established, and standard monitoring probes (electrocardiography, noninvasive blood pressure, and pulse oximeter) were applied. As a premedication, midazolam 2 mg IV was given to all patients, and all patients were hydrated with 500 mL of 0.9% sodium chloride solution IV before induction. A standardised epidural catheter insertion protocol was followed in all the study groups. The inferior border of the scapula (T7) landmark was used to identify the T9 - 10 level (Teoh et al. 2009). A 20-gauge, open-tip epidural catheter was inserted into the T9 - 10 interspace and advanced 5 cm into the epidural space while patients were in the setting position. A total volume of 10 mL was injected; 5 mL of 0.125% bupivacaine hydrochloride was mixed with each of the study doses and diluted in 5 mL of preservative-free 0.9% sodium chloride by the hospital pharmacist. We did not use the test dose of 0.5% lidocaine or 1:200,000% epinephrine and only used Dogilotti’s principle for confirmation of correct epidural catheter placement (Wilson 2007; Ikĺ 1950). If there was no rapid onset within 1–2 min of neuraxial sensory block, which was observed only during the intrathecal delivery of the local anaesthetic, the placement of the epidural catheter was activated. After 30 min, the sensory block around the level of T8-T10 was tested by pinprick. General anaesthesia was then induced using 2 mg/kg propofol and 2 mcg/kg fentanyl. Endotracheal intubation was performed with rocuronium (0.5 mg/kg), and general anaesthesia was maintained with sevoflurane and rocuronium (0.2 mg/kg on demand). Heart rate and mean arterial blood pressure (MAP) were kept within 20% of the preoperative baseline values as determined by the attending anaesthesiologist. Ondansetron (4 mg) was given as an antiemetic at skin closure. At the end of the surgery, muscle relaxation was reversed by treatment with 0.05 mg/kg neostigmine and 0.01 mg/kg atropine. After extubation, the patients were admitted to the post-anaesthesia care unit (PACU) until they fully recovered from anaesthesia.

Thereafter, the patients were transferred to the ICU, and standardised close monitoring was performed for 72 h to detect early or late respiratory depression. The same doses of epidural morphine for each patient were administered at 24 h after surgery and 48 h after surgery. Administration of epidural and IV morphine at the same time increases the risk of respiratory depression and apnoea, confusion, and haemodynamic complications. Therefore, the maximum milligrams of daily IV-PCA morphine given for each patient in all study groups hours was individually delivered according to this simple equation: (100—patient age), following a previous study for major abdominal surgery (McKeown 2015).

### Statistical analysis

The sample size was calculated using G* Power software 3.1.9.2 considering a study power of 80% and a significance level of 0.05 to detect an effect size of 0.75, to interpret large effects in gerontology and recruit larger samples (Brydges 2019), using one-way ANOVA test/t-test. Assuming total IV-PCA morphine consumption is the main outcome variable, and the maximum total IV-PCA morphine consumption was 12 ± 16 mg while in healthy controls was 70 ± 35 mg according to previous studies (Akbas et al. 2021; Tani et al. 2021; Brixel et al. 2021), the estimated minimum required sample size, after assuming 33% dropouts, was 88 patients (22 patients in each group).

Data were analysed using IBM SPSS statistics version 24 for Windows 10. A value of *P* < 0.05 was considered significant. The data were tested for normality using the Shapiro‒Wilk test. Normally distributed variables such as age and BMI are presented as the means ± SDs and were compared with ANOVA. When the assumption of normality was rejected, the nonparametric Kruskal‒Wallis test for independent samples was used for comparison. Significant Kruskal‒Wallis test values were Bonferroni corrected when various time points were investigated and when multiple tests were applied. For data presented as counts and percentages, a chi-squared test was applied.

## Results

**Fig. 1:**
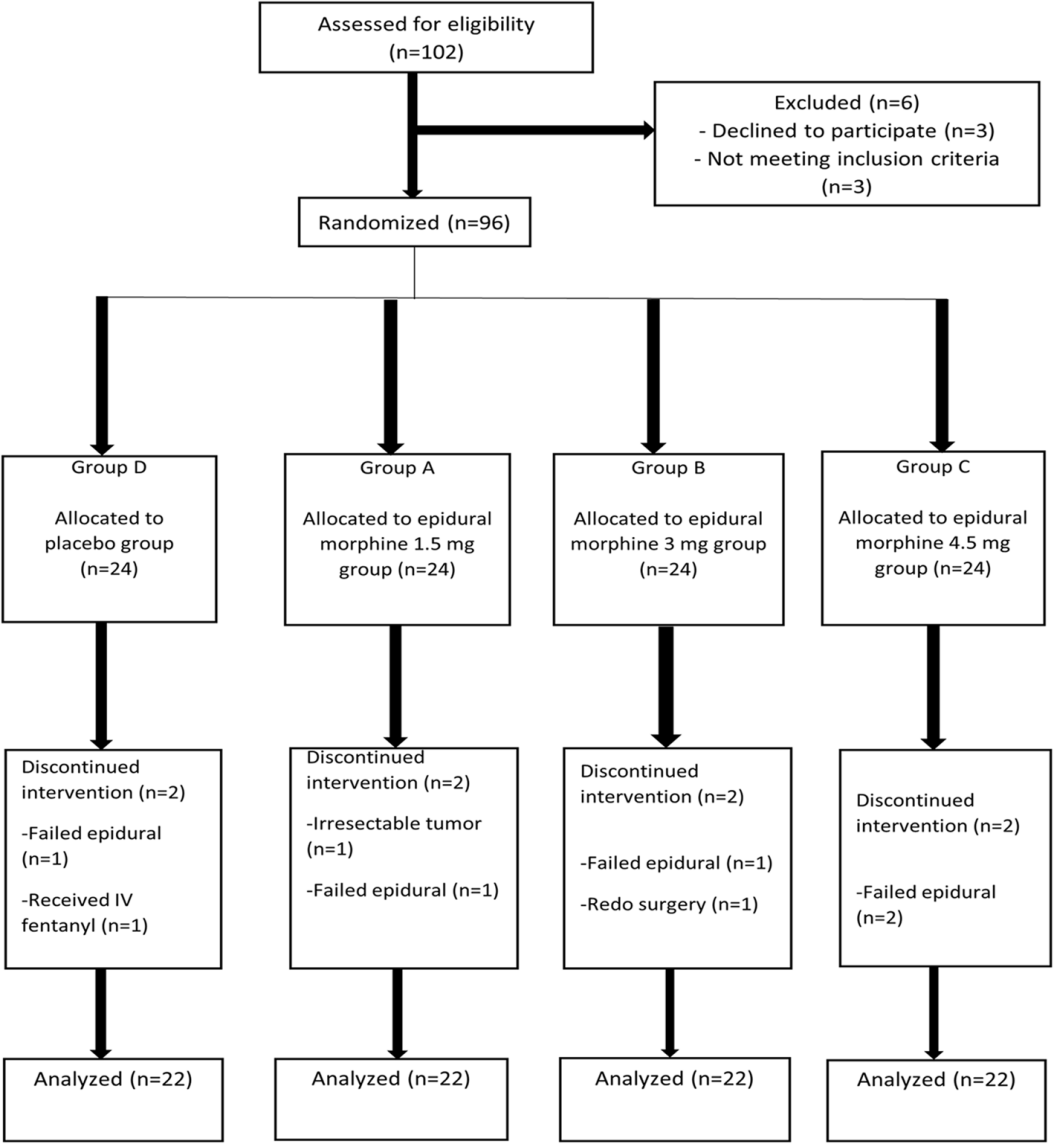
Flow chart of the patients enrolled in the study

A total of 102 patients were screened for eligibility. Three of them refused to participate in the study, and three did not meet the inclusion criteria. The remaining 96 patients were given into four equal groups. In group D (morphine 0), two patients were excluded; one patient was excluded due to failed epidural surgery, and the other was excluded due to erroneous intake of IV fentanyl; in group A (morphine 1.5 mg), two patients were excluded; one patient was excluded due to failed epidural surgery, and the other was excluded due to unresectable tumor; in group B (morphine 3 mg), two patients were excluded; one patient was excluded due to failed epidural surgery, and the other patient was excluded due to redo surgery; in group C (morphine 4.5 mg), two patients were excluded due to failed epidural surgery; therefore, 22 patients in each group remained for statistical analysis (Fig. 1). No statistically significant differences in demographic data was found between the groups (Table 1).

**Table 1:** Patient demographics

**Variable**		**1.5 mg e**pidural morphin**e**	**3 mg e**pidural morphin**e**	**4.5 mg e**pidural morphin**e**	**0 mg e**pidural morphin**e**	***P* value**
Age		66.8 (7.5)	65.3 (5.5)	65.3 (5.4)	64.3 (4.1)	0.550 ^a^
Gender	Male	8 (36.4)	6 (27.3)	10 (45.5)	7 (31.8)	0.627 ^b^
	Female	14 (63.6)	16 (72.7)	12 (54.6)	15 (68.2)	
BMI (kg/m^2^) ^*^		25 (3.4)	26.2 (3.2)	24.6 (4.1)	26.2 (3.7)	0.349 ^a^
Surgery	Total Abdominal Hysterectomy	13 (59)	13 (59.1)	12 (54.6)	15 (68.2)	0.175 ^b^
	Radical Cystectomy with ileal loop conduit	8 (36.4)	6 (27.3)	8 (36.4)	7 (31.8)	
	Radical Cystectomy with neobladder	0 (0)	0 (0)	2 (9.09)	0 (0)	
	Left Hemicolectomy	1 (4.6)	3 (13.6)	0 (0)	0 (0)	
Operative time (mins) ^**^		254.8 (57.1)	250.4 (58.5)	262.3 (67.1)	241.6 (60.3)	0.727 ^a^

Age, BMI, and operative time are presented as the mean (SD), and sex and surgery status are presented as numbers (%)

^*^kg/m^2^ = kilogram per square meter, ^**^ min = minutes

a = ANOVA, b = chi-square test

The mean total IV-PCA morphine consumption (mg) in the first postoperative 72 h was significantly higher in patients who received received epidural 0.9% sodium chloride alone compared to any of the other groups in which 1.5 mg, 3 mg, 4.5 mg epidural morphine was administered (*P* < 0.001; Bonferroni corrected). However, the difference in this variable between the 3 mg and the 4.5 mg groups was not statistically significant (*P* value = 0.191 (Bonferroni corrected) for 3 mg vs. 4.5 mg.). (Table 2). The VAS pain scores at activity of patients who received epidural morphine at doses of 3 mg and 4.5 mg were significantly lower than the other groups in the first postoperative 72 h after surgery. However, no significant difference was detected between the placebo group and the 1.5 mg group or between the 3 mg group and the 4.5 mg group (*P* value > 0.005 (Bonferroni corrected) for 0 mg vs. 1.5 mg and 3 mg vs. 4.5 mg.) (Table 2).

**Table 2:** Postoperative PCA morphine consumption and VAS score in the first 72 h

**Variable**	**1.5 mg e**pidural morphin**e**	**3 mg e**pidural morphin**e**	**4.5 mg e**pidural morphin**e**	**0 mg e**pidural morphin**e**	***P* value**
Total PCA morphine consumption 72 h	27.2 (5.6)	9.2 (3.5)	6.3 (3.3)	38.1 (4.8)	0.000^*^
Baseline VAS Score	4.6 (1.1)	2.6 (1.2)	2.2 (1.6)	5.6 (0.8)	0.000^**^
VAS Score at 2 hrs^1^	4.8 (1.2)	3 (1.3)	2 (1.6)	5.2 (0.7)	0.000^**^
VAS Score at 8 h	4.6 (1.1)	2.8 (1.1)	2.3 (1.4)	5.2 (0.6)	0.000^**^
VAS Score at 16 h	4.9 (1)	2 (1.5)	2 (1.5)	5 (0.9)	0.000^**^
VAS Score at 24 h	4.5 (0.9)	2.5 (1.1)	2.1 (1.6)	5 (0.8)	0.000^**^
VAS Score at 36 h	4.7 (1.1)	2.6 (1.1)	1.8 (1.2)	4.9 (0.8)	0.000^**^
VAS Score at 48 h	4.6 (0.9)	2.4 (1)	1.5 (1.2)	5 (1)	0.000^**^
VAS Score at 72 h	4 (1)	2 (0.8)	1.7 (1)	4.3 (1.1)	0.000^**^

The data are presented as the mean (SD), ^1^ h = hours

^*^P value = 0.191 (Bonferroni corrected) for 3 mg vs. 4.5 mg

^**^indicates a *P* value > 0.05 (Bonferroni corrected) for 0 mg vs. 1.5 mg and 3 mg vs. 4.5 mg

**Fig. 2:**
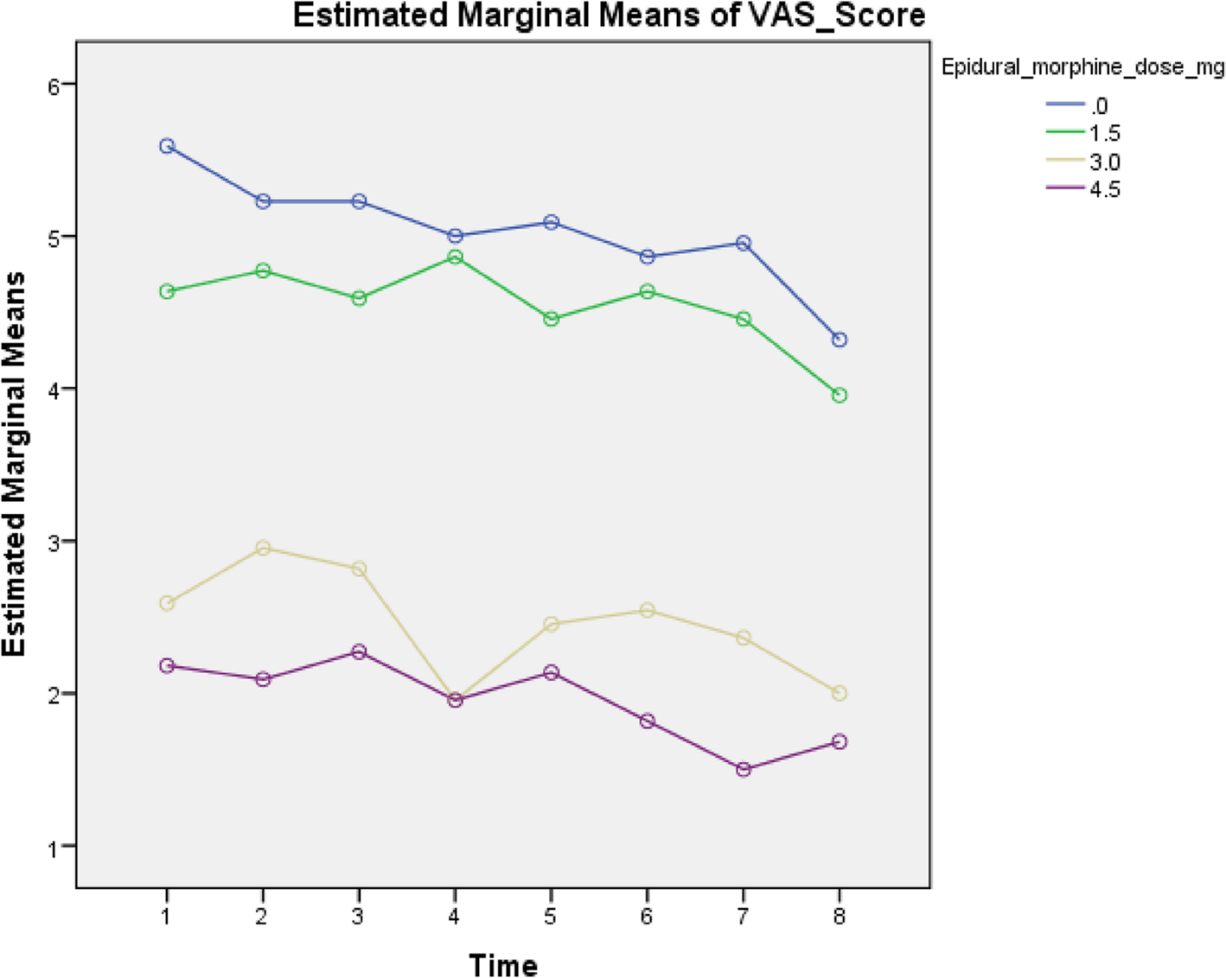
Analysis of VAS scores over time (baseline, 2 h, 8 h, 16 h, 24 h, 36 h, 48 h, 72 h) using the general linear model

Further analysis of VAS pain scores over time (baseline, 2 h, 8 h, 16 h, 24 h, 36 h, 48 h, 72 h) by the general linear model revealed statistically significant overall group differences, with a *P* value = 0.000 (Table 3). Additionally, there were significant time and group-by-time interaction effects: the VAS pain score decreased over time (baseline, 2 h, 8 h, 16 h, 24 h, 48 h, 72 h). between groups, and this decrease was greater in the 3 mg and 4.5 mg groups than in the 1.5 mg and control groups when the tests of within-subject effects and within-subject contrasts were applied (*P* value = 0.000) (Fig. 2).

**Table 3:** Analysis of VAS scores over time (baseline, 2 h., 8 h., 16 h., 24 h., 36 h., 48 h., 72 h.) using the general linear model

**Variable**	**1.5 mg e**pidural morphin**e**	**3 mg e**pidural morphin**e**	**4.5 mg e**pidural morphin**e**	**0 mg e**pidural morphin**e**	***P* value**
Vas scores over time	4.5 ± 0.2 [4.2, 4.9]	2.5 ± 0.2 [2.1, 2.8]	2 ± 0.2 [1.6, 2.3]	5 ± 0.2 [4.7, 5.4]	0.000^**^

The data are presented as the means ± SEs [95% CIs]

^**^P value > 0.05 (Bonferroni corrected) for 0 mg vs. 1.5 mg and 3 mg vs. 4.5 mg

Regarding the postoperative sedation effect, patients who received 4.5 mg of epidural morphine experienced a significant increase in the level of sedation, measured by the Ramsay sedation scale, in the first 24 h postoperatively in comparison with any of the other groups. However, none of the patients was intubated or needed mechanical ventilation. Otherwise, sedation scores were similar across the remaining groups during the first 72 h postoperatively (Table 4).

**Table 4:** Postoperative Ramsay sedation scale score, nausea score, vomiting score and pruritis score in the first 72 h

**Variable**	**1.5 mg e**pidural morphin**e**	**3 mg e**pidural morphin**e**	**4.5 mg e**pidural morphin**e**	**0 mg e**pidural morphin**e**	***P* value**
Baseline Ramsay Sedation Scale	1.9 (0.5)	2. (0.4)	3 (0.4) ^**a, b, c**^	1.7 (0.5)	0.000
Ramsay Sedation Scale 2 h	2 (0.4)	2.2 (0.4)	3 (0.4) ^**a, b, c**^	1.9 (0.5)	0.000
Ramsay Sedation Scale 8 h	2.1 (0.2)	2.1 (0.2)	2.9 (0.5) ^**a, b, c**^	2 (0.2)	0.000
Ramsay Sedation Scale 16 h	2 (0.3)	2 (0.2)	2.9 (0.5) ^**a, b, c**^	2 (0.3)	0.000
Ramsay Sedation Scale 24 h	2.1 (0.2)	2.1 (0.2)	2.5 (0.5) ^**a, b, c**^	2.2 (0.5)	0.000
Ramsay Sedation Scale 36 h	2.1 (0.2)	2.1 (0.2)	2.1 (0.5) ^**a, b, c**^	2.4 (0.5)	0.05
Ramsay Sedation Scale 48 h	2.1 (0.2)	2.1 (0.2)	2.1 (0.2)	2.1 (0.2)	1
Ramsay Sedation Scale 72 h	2.1 (0.2)	2.1 (0.2)	2.1 (0.2)	2.1 (0.3)	0.893
Nausea score	4.4 (0.8)	4.5 (0.7)	4.6 (0.7)	4.8 (0.7)	0.274
Vomiting Score	3.1 (1)	3 (1.9)	3.9 (2.1)	1.5 (1.2) ^**c, d, e**^	0.000
Pruritus Score	3.4 (1)	3.7 (1.1)	3.2 (0.8)	1.9 (1.4) ^**c, d, e**^	0.000

The data are presented as the mean (SD). a, b, c, d and e mean *P* value < 0.005 (Bonferroni corrected)

a = 4.5 mg vs. 1.5 mg, b = 4.5 mg vs. 3 mg, c = 4.5 mg vs. 0 mg, d = 3 mg vs. 0 mg, e = 1.5 mg vs. 0 mg

The postoperative vomiting and pruritus scores were significantly greater in the treatment groups that received epidural morphine than in the placebo group (*P* value < 0.005; Bonferroni corrected). However, no significant differences were detected between the 1.5 mg, 3 mg, and 4.5 mg groups. The nausea score was similar across both the placebo and treatment groups (*P* value = 0.274). (Table 4).

## Discussion

In this study, we compared the analgesic efficacy of three doses of epidural morphine (1.5 mg, 3 mg, and 4.5 mg) given as a bolus every 24 h with a placebo group in the population of older adults who underwent lower abdominal cancer surgery. A 72-h follow up period was chosen to assess the side effect of epidural morphine over a longer period enough to not miss delayed respiratory depression or extenuated sedation. The results showed that IV-PCA morphine requirements were reduced significantly with 3 mg and 4.5 mg of epidural morphine, but 3 mg of epidural morphine resulted in less sedation than 4.5 mg of epidural morphine. Further comparison did not detect any significant difference in IV-PCA morphine requirements between the 3 mg and 4.5 mg groups. Pain scores were significantly decreased with epidural morphine doses of 3 mg and 4.5 mg compared to 1.5 mg and placebo groups. However, 4.5 mg of epidural morphine did not provide any analgesic advantage when it was compared with the 3 mg group. Surprisingly, there was no statistically significant difference between the 1.5 mg epidural morphine group and the placebo group in terms of the reduction in pain intensity in older adults.

Comparable to our previous findings, Palmer et al. (2000), who studied single postoperative doses ranging from 0 to 5 mg after cesarean section operations, determined that the degree and duration of analgesia increase as the dose of epidural morphine increases over the range of 0 to 3.75 mg, and analgesia (as also measured by IV-PCA morphine consumption) did not further increase beyond a dose of 3.75 mg. Furthermore, the smallest dose in this series, 1.25 mg, had a modest IV-PCA morphine sparing effect that persisted through a 24-h period. Fuller et al. (Fuller et al. 1990) retrospectively reviewed 4880 women who received single postoperative doses ranging from 2 to 5 mg after cesarean section operations and concluded that 3 mg is an “adequate” dose based on the duration of analgesia. Although postoperative pain after lower abdominal cancer surgery is reported to be more severe than postoperative pain after caesarean section surgery, 3 mg of epidural morphine was also adequate in our study, mostly because older adults appear to be more sensitive to epidural morphine than young women are (Rawal et al. 1981). On the other hand, 3 mg epidural morphine is considered slightly risky compared to a previously published study in Anaesthesia and Analgesia addressing older adult patients who recommended that doses beyond 2 mg should be avoided irrespective of the type of surgery (Rawal et al. 1981). However, we believe that this study involved different types of surgeries, such as orthopedic and perineal surgeries, where doses greater than 2 mg might be unnecessary. Mugabure et al. suggested that the optimal single-shot epidural morphine dose could be 2.5–3.75 mg for the first 24 h after surgery (Mugabure 2012).

We understand that a single dose of epidural morphine usually provides analgesia lasting up to 24 h. Moreover, epidural morphine should be given as a repeated bolus rather than as a continuous infusion to avoid supraspinal action-related side effects. Therefore, epidural morphine was administered via the bolus method without postoperative admixture with local anaesthetics to ensure that postoperative analgesia was only maintained by morphine without confounding factors. In addition, the co-injection of epidural local anaesthetics should be based on the nature of the expected complications. For example, it would be wiser to avoid epidural injection of local anaesthetics in patients who are sensitive to hypotension such as older adults with hypertension or vascular stiffness to achieve early mobilisation and stable blood pressure. On the other hand, we need to clinically know the highest safe limit of epidural injection of morphine alone without compromising the respiratory function in the context of abdominal surgery.

Our findings indicate that increasing the dose of epidural morphine is correlated with increased level of sedation. Patients who received 4.5 mg of epidural morphine were found to higher scores on Ramsay Sedation Scale in the first 24 h after surgery, with non-significant analgesic superiority over those in the 3 mg epidural morphine group. Previous studies on epidural morphine analgesia did not evaluate its effects on the level of sedation, but it is highly important to consider this point in the current study because morphine causes significant early or late respiratory depression in this age group (Jarzyna et al. 2011). Furthermore, opioid-induced respiratory depression (OIRD) may be less commonly reported in clinical settings because it is often miscategorised as simply sedation or bradypnea (Dahan et al. 2010).

Following Palmer and his colleagues, we did not find any relationship between the incidence or severity of vomiting or pruritus and the dose of epidural morphine because all the treatment groups had higher nausea and vomiting scores than did the placebo group. This is the opposite of the dose-related vomiting and pruritus described after intrathecal morphine administration, (Palmer et al. 1999) but this could be explained by the smaller dose range studied compared to other studies. If a wider scale for scoring side effects had been used (rather than the 0–2 scale), it is more likely that subtle differences may have been detected among the groups. However, the use of this scale decreases the possibility of subjective interpretation and makes it more reproducible. Furthermore, such minor differences would not be clinically significant.

It is also worth noting that the least level of sedation was observed among patients in the placebo group. In addition, those patients had significantly lower incidence of vomiting and itching. This highlights the fact some argue that paracetamol could be the first-line analgesic for acute pain in older adults, while opioids should be cautiously administered for moderate to severe acute pain (Abdulla et al. 2013).

OIRD and postoperative nausea and vomiting (PONV) are now more common than previously reported findings in inpatient settings (Oderda et al. 2019). It has also been reported that epidural morphine is associated with a significant increase or no decrease in postoperative nausea (Bonnet et al. 2010; Stannard and Jang 2020). In contrast, our results showed no relationship between postoperative nausea and epidural morphine, as no statistically significant difference was found in the nausea score across the study groups. The mechanism of epidural morphine-induced nausea and vomiting is assumed to involve the activation of opioid receptors in the chemoreceptor trigger zone of the fourth ventricle caused by cephalad migration of epidural morphine. However, a different unknown mechanism is more likely to exist in most patients. For this reason, initial treatment of nausea and vomiting with an antiemetic rather than an opioid antagonist may be more effective in patients who have received epidural morphine.

## Strength points and limitations

The main strengths of this study are that it is highly reproducible and addresses the daily clinical aspects of epidural morphine in older adults. Due to the vulnerability of older adults, we included only ASA I and II patients to assess the balancing dose of epidural morphine versus age only. There might have been an underestimation of the ASA physical status classification in this study, especially for two patients with sigmoid cancer who were classified as ASA I. This is because the classification has moderate inter-rater variability, which is inherent to the subjectivity characteristics (Karim 2021). Such subjectivity may result in overlooking the systemic nature of a sigmoid cancer that was discovered accidentally during routine checkups in an apparently healthy patients. The relationship between the dose of epidural morphine and common comorbidities in this age group should be investigated.

Moreover, this study is limited by its focus on lower abdominal cancer surgeries only; additionally, it is a single-center study. Therefore, the generalizability of our results is limited. Future large studies on different types of surgeries are needed. This study was more focused on the dose versus clinical outcomes and did not fully investigate the pharmacokinetics-pharmacodynamics (PK-PD), which should be examined in future studies. We could not include patient and the public in developing this research due to their limited interest in the educational aspects of our study. Therefore, it is internationally encouraged to include this age group in the design of future trials to shape further research questions.

## Conclusion

We concluded that epidural morphine 3 mg as a bolus every 24 h with add on IV patient control analgesia (PCA) morphine, set to deliver 1.5 mg boluses on demand without background infusion with a lockout period of 45 min, could achieve effective and adequate analgesia lasting up to 72 h postoperatively without increasing in the level of sedation or other side effects in older adults after a lower abdominal cancer surgery.

## Supplementary Information

Supplementary Material 1

## Data Availability

The datasets generated during and/or analysed during the current study are available from the corresponding author upon reasonable request. The data are not publicly available because they contain information that could compromise research participant privacy/consent.

## Abbreviations

PK-PD: Pharmacokinetics-pharmacodynamics
VAS: Visual Analogue Scale
PCA: Patient-controlled analgesia
IV: Intravenous
PACU: Post-Anaesthesia Care Unit
ICU: Intensive care unit
OIRD: Opioid-induced respiratory depression
PONV: Postoperative nausea and vomiting
